# Different antibiotic growth promoters induce specific changes in the cecal microbiota membership of broiler chicken

**DOI:** 10.1371/journal.pone.0171642

**Published:** 2017-02-21

**Authors:** Marcio C. Costa, Jose A. Bessegatto, Amauri A. Alfieri, J. Scott Weese, João A. B. Filho, Alexandre Oba

**Affiliations:** 1 Department of Veterinary Preventive Medicine, Universidade Estadual de Londrina, Londrina, Paraná, Brazil; 2 Department of Pathobiology, Ontario Veterinary College, University of Guelph, Guelph, Ontario, Canada; 3 Department of Animal Science, Universidade Estadual de Londrina, Londrina, Paraná, Brazil; University of Minnesota, UNITED STATES

## Abstract

Antimicrobials are sometimes given to food animals at low doses in order to promote faster growth. However, the mechanisms by which those drugs improve performance are not fully understood. This study aimed to investigate the impact of zinc bacitracin (55g/ton), enramycin (10g/ton); halquinol^®^ (30g/ton); virginiamycin (16,5g/ton) and avilamycin (10g/ton) on the cecal microbiota of broiler chicken, compared to a control group. Six hundred and twenty four chicks (Cobb 500) arriving to an experimental unit were randomly assigned into each treatment with four repetitions per treatment. The cecal content of 16 animals per treatment (n = 96) was used for DNA extraction and sequencing of the V4 region of the 16S rRNA gene using Illumina technology. The use of antimicrobials induced significant changes in membership but not in structure of the cecal microbiota compared to the control group, suggesting a greater impact on the less abundant species of bacteria present in that environment. Halquinol was the only drug that did not affect microbial membership. Firmicutes comprised the major bacterial phylum present in the cecum of all groups. There was no statistical difference in relative abundances of the main phyla between treated animals and the control group (all P>0.05). Treatment with enramycin was associated with decreased richness and with lower relative abundance of unclassified Firmicutes, *Clostridium XI*, unclassified Peptostreptococcaceae (all P<0.001) and greater abundance of *Clostridium XIVb* (P = 0.004) and *Anaerosporobacter* spp. (P = 0.015), and treatment with bacitracin with greater relative abundance of *Bilophila* spp. (P = 0.004). Several bacterial genera were identified as representative of usage of each drug. This study used high throughput sequencing to characterize the impact of several antimicrobials in broiler chicken under controlled conditions and add new insights to the current knowledge on how AGPs affect the cecal microbiota of chicken.

## Introduction

Antibiotic growth promoters (AGPs) have been widely used to improve performance of food animals. Antimicrobials are given to broiler chicken in order to control diseases such as necrotic enteritis caused by *Clostridium perfringens*, and also to promote faster growth and improve conversion rates [1–3]. The effects of those drugs are not fully understood, but the potential of the intestinal microbiota in increasing feed efficiency has been shown [4–7].

This practice has recently raised concerns regarding emergence of antibiotic resistant strains of bacteria that could potentially spread to humans [8]. Indeed, AGPs usage has been banned in the European Union and there is increasing pressure for stricter regulations in North America [9].

The intestinal microbiota has been shown to have a tremendous influence on host health and disturbances in its balance (dysbiosis) have been associated with various diseases [10]. While several factors, such as diet, environment and genetics can induce changes in the intestinal microbiota, the use of antimicrobials is one of the most important [11]. The different spectrum of selection depending on the active ingredients present in each compound should induce predictable changes on the intestinal microbiota [12]. However, the in order to adequately address those changes, controlled environmental conditions should be used for the characterizations of changes induced by those drugs.

Changes in the cecal environment are of importance since cecal bacteria are responsible for food fermentation and short chain fatty acids (SCFA) production in chickens [13–15]. Therefore, a better characterization of how AGPs impact the cecal microbiota of chickens could be the keystone for the development of alternative methods to improve growth efficacy in this species [16].

The effects of AGPs on the chicken microbiota have recently been investigated [3,17,18]. However, since several factors can impact the intestinal microbiota, comparison between studies is difficult and in order to adequately compare the effect of different drugs molecules, it is essential to secure that experimental conditions are rigorously controlled. To date, most studies using high throughput sequencing have compared a limited number of drugs, with special emphasis given to virginiamycin and bacitracin [3,19–21].

This study was designed to test the hypothesis that different antimicrobial drugs would differentially affect the cecal microbiota of broiler chicken.

## Materials and methods

### Study design

This study was approved by the University of Londrina’s Animal Care and Use Committee (process number: 10482014.57).

Six hundred and twenty four 2 day-old chicks (Cobb 500) arriving to the poultry farm of the experimental unit of the Londrina University (Londrina, Paraná State, Brazil) were allocated into cages of 1.45 x 1.45 m (26 animals per cage). Cages were randomly assigned into six treatment groups with four repetitions per treatment: control (no antibiotic), zinc bacitracin (55g/ton), enramycin (10g/ton); halquinol (30g/ton); virginiamycin (16,5g/ton) and avilamycin (10g/ton). All animals received a standard diet recommended for this breed from 1 to 21 days of life and another from 22 to 42 days of life, constituted mainly of corn (63%), grinded soy (28%) and soy oil (5%) (S1 Table). Antimicrobials were given to animals during the whole period of the trial.

Chicks were vaccinated at the hatchery against Marek’s disease and at the experimental unit when were 10 day-old against gumboro (Bursine 2, Zoetis, New Jersey USA). Litter consisted of wood shavings was added to the cages to a height of 6 cm. Approximately 300 grams of litter from a commercial farm were mixed to the clean litter in order to increase challenge with disease strains present in the field.

Weight gain and feed intake were recorded and feed conversion and viability calculated within each group. Four animals per cage (n = 16/group; 96 total) were randomly selected at the time of slaughter (43 days) and had their cecal contents collected into sterile plastic tubes that were promptly refrigerated (for a maximum of 1.5 hour) and frozen at -80°C until DNA extraction. Chickens were rendered unconscious by electrical stunning just before slaughtering by exsanguination.

### DNA extraction and sequencing

DNA was extracted with a commercial kit (E.Z.N.A. Stool DNA Kit, Omega Bio-Tek) according to the manufacturer’s instructions. The V4 region of the gene 16S rRNA was amplified by PCR using the forward S-D-Bact-0564-a-S-15 and the reverse primers S-D-Bact-0785-b-A-18 [22] containing an overlapping region of the Illumina sequencing primers. PCR was carried in two steps: first, 2.5μL of DNA were added to a mixture containing 9μL of water, 12.5μL of Kapa 2X ReadMix (Kapa Biosystems. MA) and 0.5μL of each 16S primer (10 pmol/μL). The reaction was carried according to the PCR conditions: 3 min at 94°C for denaturing, and 26 cycles of 45s at 94°C for denaturing, 60s at 53°C for annealing and 90s at 72°C for elongation followed by a final period of 10 min at 72°C and kept at 4°C. PCR products were purified with 20 μL of Agencourt AMPure XP (Beckman Coulter) magnetic beads and eluted in 52.5 μl Tris buffer (10mM, pH 8.5). The second PCR was carried by adding 4μL of the purified product to a mixture with 9.6μL of water, 20μL of 2X Ready Mix and 3.2μL of each Illumina index primer (2.5pmol/μL), which was submitted to the following PCR conditions: 3 min at 94°C, and 7 cycles of 45s at 94°C, 60s at 50°C and 90s at 72°C and a final period of 10 min at 72°C and kept at 4°C. A second purification was performed by using 40μL of AMPure beads and eluting samples with 32μL of Tris buffer (10mM, pH 8.5). Sequencing was performed with an Illumina MiSeq platform with the V2 reagents kit for 250 cycles from each end at the Genomics Facility of the University of Guelph.

Data were made publicly available at the NCBI Sequence Read Archive under accession number SRP096720.

### Statistical analysis

Bioinformatic analysis was performed using the software mothur (v.1.36.1) following the standard operational protocol recommended by Kozich et al. [23]. In short, after data clean up sequences were assigned into phylotypes at the genus level (94% similarity) with taxonomic classification obtained from the Ribosomal Database Classifier (March 2012) [24].

Relative abundances of the main phyla and genera (abundance >1%) and the Firmicutes:Bacteroidetes (F:B) and Firmicutes:Proteobacteria ratios (F:P) found in each treatment were represented by column charts. The effect of treatment on each variable was investigated using the ANOVA on ranks with the Kruskal-Wallis non-parametric test and the Tukey test for multiple comparison correction considering a P<0.010 as statistically significant to decrease false discovery rate. The ANOVA with Tukey’s test (considering a P<0.05 as significant) was used to investigate interactions between treatments and performance indexes.

In order to decrease bias caused by non-uniform sequence numbers, a subsample from the main dataset was used for alpha and beta diversity analyses and the Good´s coverage bootstrap was calculated in order to ensure that the cutoff adopted was representative of original samples. Richness was estimated by the Chao index and by the number of observed genera. The Shannon and the Simpson’s indices were used to estimate diversity. The effect of treatment on those variables was investigated using the ANOVA on ranks with the Kruskal-Wallis non-parametric test.

Community membership (that considers the different genera present in each sample) and structure (that considers the different genera and their evenness in each sample) were addressed respectively by the classic Jaccard and by the Yue and Clayton index [25]. The similarity between community membership and structure present in each sample was represented by dendrograms visualized with FigTree (v1.4.2) (http://tree.bio.ed.ac.uk/software/figtree/), and by the Principal Coordinate Analysis (PCoA) with two dimensions. The Parsimony test and the analysis of molecular variance (AMOVA) were used for statistical comparison of communities’ membership and structure between the groups, and the Benjamini-Hochberg for multiple comparisons adjustment using a false discovery rate of 0.20.

The “indicator analysis” implemented in mothur [26] was used with a cutoff of 0.01 in order to identify the most representative bacterial taxa present within each group. In addition, the linear discriminant analysis (LDA) Effective Size (LefSe) was used to detect meaningful biological differences between treatments [27]. P values <0.05 and logarithmic LDA scores higher than 2.0 were considered as significant.

## Results

A total of 5,695,309 good quality reads were used for the analysis, from which 15,510 reads per sample were randomly subsampled to normalize sequence numbers. The subsampling yielded coverage of 99.9%, indicating that it was representative of the total population.

### Alpha diversity

Average and standard deviation of alpha diversity indices found in each group are presented in Table 1. The statistical analysis revealed that only enramycin was associated with decreased richness estimated by the number of observed genera (P = 0.007) and by the Chao index (P = 0.031) compared to controls. There was no statistical difference between the other treatment groups regarding the number of observed genera, estimated richness (Chao), or diversity (Simpson and Shannon indices).

**Table 1:** Average and standard deviation (in brackets) of the number of different genera and results of Chao, Simpson and Shannon indexes present in the cecum of broiler chicken after treatment with different antibiotic growth promoters.

	# genera	Chao	Simpson	Shannon
**Avilamycin**	92.94 (5.32)	108.84 (6.88)	10.34 (1.55)	2.88 (0.11)
**Bacitracin**	93.67 (2.91)	109.67 (4.92)	10.05 (2.17)	2.84 (0.16)
**Halquinol**	94.04 (8.78)	108.31 (11.86)	9.92 (2.28)	2.80 (0.19)
**Enramycin**	87.48 (4.32)	102.11 (7.85)	10.25 (2.11)	2.83 (0.16)
**Virginiamycin**	91.73 (4.09)	107.62 (5.32)	9.25 (1.80)	2.79 (0.16)
**Control**	93.13 (7.04)	110.69 (8.77)	9.89 (2.21)	2.79 (0.19)

### Beta diversity

**Fig 1:**
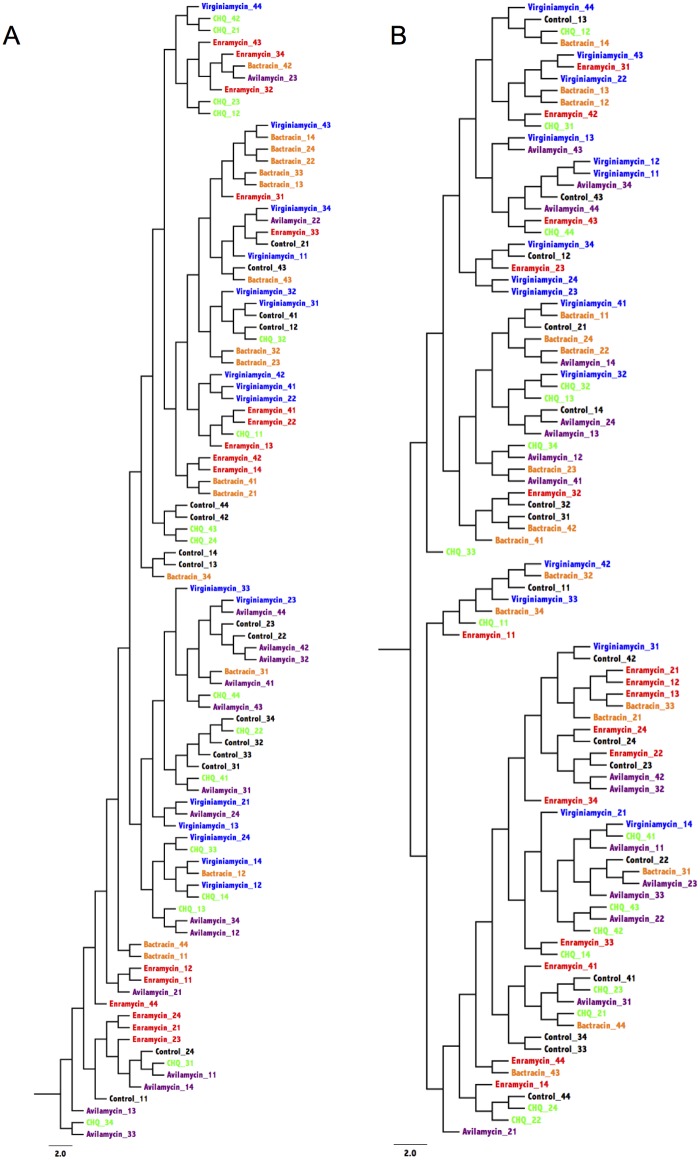
Dendrograms representing the similarity between membership (A) and structure (B) of bacterial communities found in cecum of broiler chicken treated with zinc bacitracin (orange), enramycin (red); halquinol (green); virginiamycin (blue), avilamycin (purple) and in a control group (black). CQH: Halquinol.

**Fig 2:**
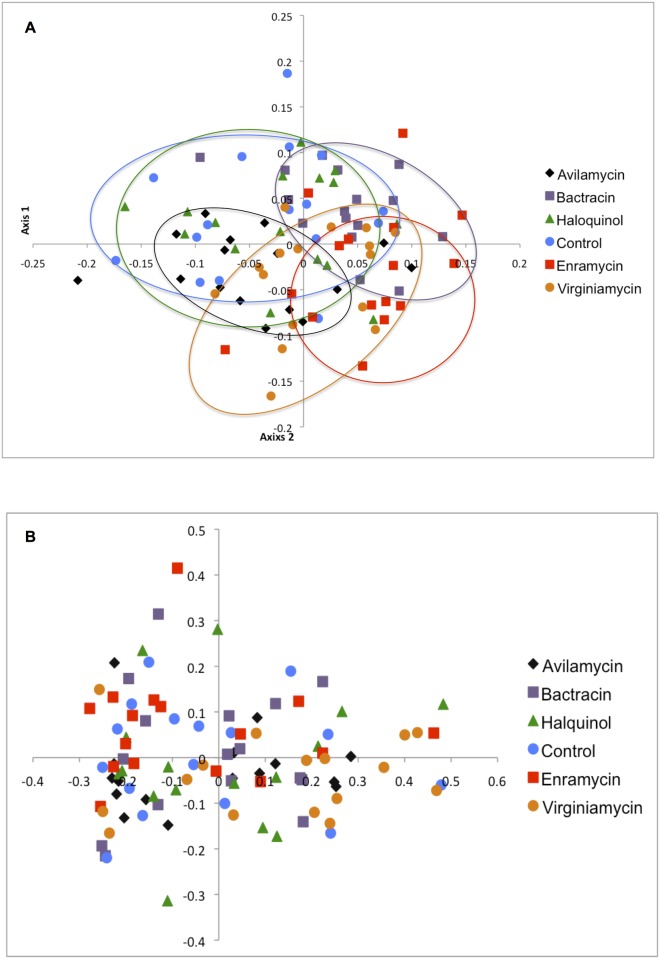
Principal Coordinate Analysis (PCoA) representing the similarity between membership (A) and structure (B) of bacterial communities found in cecum of broiler chicken treated with several antibiotic growth promoters.

The similarity between bacterial communities present in each sample is represented by dendrograms in Fig 1 and Principal Coordinate Analysis (PCoA) in Fig 2. Fig 2A represents microbial membership present in each sample and is indicative that each antimicrobial drug tended to have a specific effect on the selection of cecal species, evidenced by the formation of clusters according to the different treatments. Noteworthy is the overlapping between controls and animals treated with halquinol, indicating a limited apparent impact of this drug on the microbiota. The lack of action on microbial community structure is evident from Fig 2B, with overlapping of samples from the different treatments.

The effect of antimicrobials on microbial membership is further evidenced by Parsimony and AMOVA results (Table 2). Halquinol was the only drug that did not affect microbial membership compared to the control group. Interestingly, treatment with antimicrobials did not affect microbial structure in the cecum of any of the studied groups. This, along with the changes caused in membership, suggests that those drugs had a greater impact on rare species present in that environment.

**Table 2:** P values obtained from the comparison of microbial membership (gray background) and structure (white background) using the Parsimony and AMOVA tests.

**Parsimony**						
	Avilamycin	Bacitracin	Halq	Enramycin	Virginiamycin	Control
Avilamycin		0.380	0.625	0.012	0.356	0.365
Bacitracin	**0.004**		0.340	0.358	0.357	0.967
Halquinol	0.356	**0.056**		0.362	0.366	0.852
Enramycin	**0.003**	**0.014**	**0.010**		0.151	0.964
Virginiamycin	**0.045**	**0.050**	0.351	**0.006**		0.628
Control	**0.041**	**0.046**	0.134	**0.007**	**0.050**	
**AMOVA**						
	Avilamycin	Bacitracin	Halq	Enramycin	Virginiamycin	Control
Avilamycin		0.231	0.551	0.098	0.058	0.724
Bacitracin	**<0.001**		0.573	0.388	0.042	0.739
Halquinol	**<0.001**	**<0.001**		0.147	0.142	0.809
Enramycin	**<0.001**	**<0.001**	**<0.001**		0.018	0.212
Virginiamycin	**<0.001**	**<0.001**	**0.002**	**<0.001**		0.065
Control	**<0.001**	**<0.001**	0.142	**<0.001**	**<0.001**	

Statistically significant results after the Benjamini-Hochberg adjustment are in bold.

Halq: Halquinol

### Relative abundances

**Fig 3:**
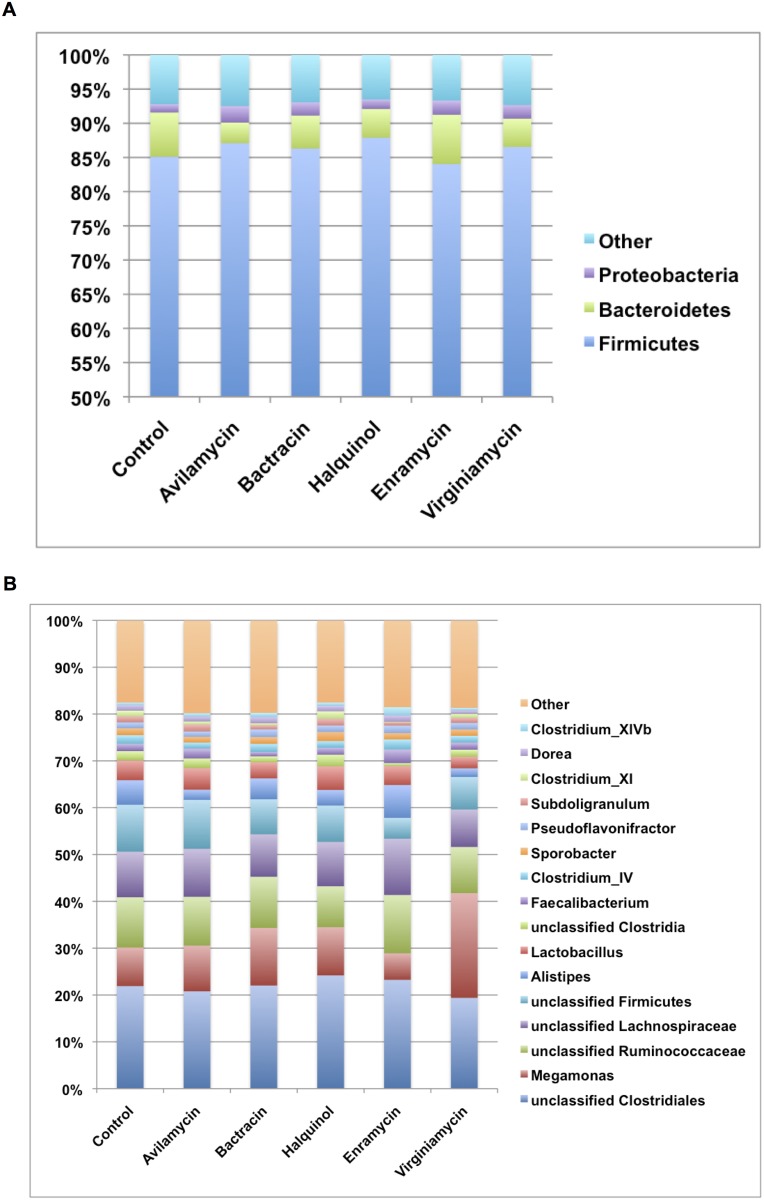
Relative abundances at the phylum (A) and genus (B) level of the main bacteria found in the cecum of broiler chicken treated with zinc bacitracin, enramycin; halquinol; virginamycin, avilamycin and in a control group.

Relative abundances of the main phyla and genera are presented in Fig 3 and S1 Fig. Firmicutes comprised the major bacterial phylum present in the cecum of all groups. Along with Bacteroidetes and Proteobacteria, these three phyla accounted for more than 90% of all sequences. There was no statistical difference in the relative abundances between treated animals and the control group (all P>0.05).

Compared to the control group, animals treated with enramycin had statistically lower relative abundance of unclassified Firmicutes, Clostridium XI, unclassified Peptostreptococcaceae (all P<0.001) and greater abundance of Clostridium XIVb (P = 0.004) and *Anaerosporobacter* (P = 0.015). In addition, animals treated with bacitracin had greater abundance of *Bilophila* (P = 0.004) compared to controls.

**Fig 4:**
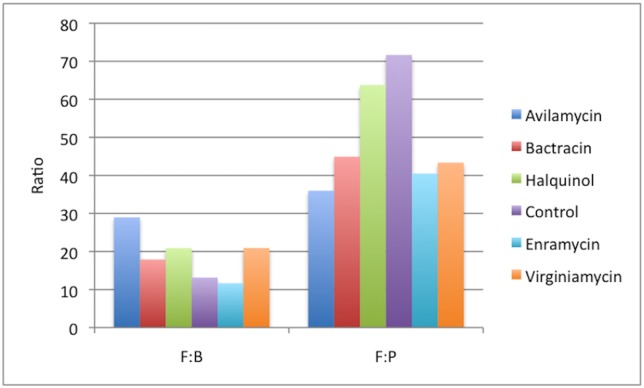
Firmicutes to Bacteroidetes (F:B) and Firmicutes to Proteobacteria (F:P) ratios present in the cecum of broiler chicken treated with different antibiotic growth promoters.

The Firmicutes:Bacteroidetes and Firmicutes:Proteobacteria ratios were calculated within each treatment group and are presented in Fig 4. Firmicutes:Bacteroidetes ratio did not differ between groups (all P>0.05), but all groups treated with AGPs had numerically lower Firmicutes:Proteobacteria ratios compared to controls.

### Specific taxa associated with each treatment

The taxa considered more likely to be representative of each group (P>0.001) as per the indicator analysis are presented in Table 3. No significantly discriminative features could be identified using the LefSe analysis.

**Table 3:** Bacterial taxa found to be significantly associated with as representative of each treatment as per the indicator analysis.

Treatment	Taxa
Avilamycin	Unclassified Sutterellaceae
Bacitracin	Faecalibacterium, *Anaerofilum*, *Hydrogenoanaerobacterium*
Halquinol	Turicibacte, *Clostridium*_XI, unclassified Firmicutes
Enramycin	*Clostridium*_XlVb, *Anaerosporobacter*, *Planococcaceae*, *Holdemania*, *Eubacterium*
Virginiamycin	*Meniscus*, *Lachnospira*, *Megamonas*
Control	*Cronobacter*, unclassified Hyphomonadaceae

Table 4 contains data from the performance indexes calculated for each treatment. avilamycin was the only drug associated with higher weight gain and feed conversion.

**Table 4:** Weight gain (in grams), feed conversion and viability (in percentage) observed in chickens treated with different antibiotic growth promoters.

Treatment	Weight gain (g)*	Feed conversion*	Viability (%)
Control	2915 ^ab^	1,70 ^a^	98,07
Avilamycin	3066 ^a^	1,61 ^b^	97,11
Bacitracin	2841 ^ab^	1,70 ^a^	97,11
Halquinol	2668 ^b^	1,75 ^a^	93,27
Enramycin	2707 ^b^	1,71 ^a^	89,42
Virginiamycin	2723 ^b^	1,74 ^a^	95,19

* different letters in the same column represent significant difference between treatments.

## Discussion

None of the antibiotics selected for this study caused significant changes in cecal community structure (related to the genera comprising a community and how they are distributed) compared to the control group, but did affect microbial membership (the different genera present in the cecum). Thus, while there were significant changes in the specific bacterial members that were present (Jaccard index), the lack of an overall impact on the microbial structure (Yue and Clayton index) would suggest that changes affected rare members of the microbiota. This could be related to the fact that most evident changes would be expected to happen at earlier ages and in the most proximal compartments of the intestinal tract [18]. Nevertheless, these findings are particularly important for a better understanding of how AGPs affect cecal bacterial populations and might be used in the future for microbiota manipulation.

Although microbial membership was significantly different from controls regardless the statistical test applied (Table 2), clustering of samples according to the different treatments was not as strong as it has been shown with the use of therapeutic doses [28]. Yet, some clustering can still be noticed in the principal coordinate analysis (Fig 2A).

Halquinol was the only drug that did not alter significantly the membership of the cecal microbiota in any detectable way. The similarity between animals treated with the drug and controls is evidenced by the overlapping of samples observed in Fig 2A. However, at this point, it is not clear whether this “lack of action” is desirable or not. Guasti [26] reported lower weight gain in chicken receiving halquinol compared to groups that also received avilamycin and a prebiotic, which could indicate that this drug may not adequately select species associated with better efficiency for harvesting feed energy. In agreement with those results, weight gain from animals treated with halquinol in the present study did not differ from the control group, but further studies are necessary before solid conclusions can be made.

The group treated with avilamycin was the only to have statistically greater weight gain and better feed conversion compared to controls. Although no genera of biological significance could be identified using the LefSe analysis, but an unclassified Sutterellaceae was associated with the use of this drug pointed by the indicator analysis and may be of importance for the development of new alternative approaches to decrease the use of antimicrobials for growth promotion, such as probiotics [3,29].

Enramycin was associated with decreased diversity and greatly affected the relative abundance of several genera compared to controls. Pedroso et al. [30] also reported changes in composition of the intestinal microbiota of chicken treated with this drug, but the characterization of those changes was not possible since molecular fingerprinting was used in that study.

Fasina et al. [3] showed that treatment with bacitracin suppressed experimental infection with *C*. *perfirngens*, since treated animals presented lower abundance of the Clostridiaceae family. This drug has also been reported to deplete members of the Lactobacillaceae family [3,20,21], but the same trend could not be observed in the present study.

Virginiamycin was associated with enrichment of *Faecalibacterium* and *Lactobacillus* spp. and with decreased diversity in the cecal microbiota [21]. Also, the use of this drug along with monensin was reported to cause depletion of Firmicutes compared to a control group [31]. However, those findings could not be confirmed by our results and that might be related to differences in diet, geographical location and methodological analyses.

Greater Firmicutes:Bacteroidetes ratios have been associated with bacterial profiles with higher capacity of energy harvesting [7,32,33]. Firmicutes are also reported to be the main phylum in commercial broilers, while free range chicken seems to present with increased Bacteroidetes and Proteobacteria [34]. Interestingly, the group with the highest Firmicutes:Bacteroidetes ratio in this study was the only one to gain statistically more weight. However, differences in ratios between groups were not significant, and further investigations are required before any assumption can be made.

Firmicutes, along with Bacteroidetes and Proteobacteria comprised the major bacterial phyla present in the cecum of all groups. Despite selection bias towards Verrucomicrobia detection usually observed with sequencing of the V4 region of the 16S gene, this phylum was not amongst the most abundant in this study, which further supports the findings of other researchers [35,36]. While results of this study is in agreement with other reports that showed *Clostridium*, *Lactobacillus* and *Ruminococcus* spp. as the most abundant genera in the chicken cecum [31,37], the high abundance of *Megamonas* spp. was not expected. This genus has been recently classified and is considered a commensal organism present in the intestinal tract of mammals and birds [38].

Since the 1940’s low doses of antimicrobials have been given to food animals to increase weight gain, but the mechanisms of how this drugs favor the increase in productivity are still under investigation. The role that the intestinal microbiota plays in this process has been demonstrated [4] and further studies should focus on gut microbiota manipulation in order to improve productivity and animal health.

The results of this study add new insights to the current knowledge on how AGPs affect the cecal microbiota of chicken.

## Conclusions

The use of several antimicrobials at growth promotion doses caused significant changes in the cecal microbial membership of broiler chicken, but not in microbial structure, suggesting that those drugs have a stronger impact on the rare species of bacteria present in that environment.

## Supporting information

S1 Table Feed formulations given to chickens from 1 to 21 and 22 to 42 days of life. (DOCX)

S1 Fig Individual representation of the relative abundances at the phylum (A) and genus (B) level of the main bacteria found in the cecum of broiler chicken treated with zinc bacitracin, enramycin; halquinol; virginamycin, avilamycin and in a control group. (TIFF)
