# Supplementary material for: Different antibiotic growth promoters induce specific changes in the cecal microbiota membership of broiler chicken
Source: PLoS One. 2017 Feb 21;12(2):e0171642. doi: 10.1371/journal.pone.0171642 (PMC5319738; doi:10.1371/journal.pone.0171642)
Supplement: S1 Table — (DOCX) [file pone.0171642.s001.docx]

**S1 Table.** Feed formulations given to chickens from1 to 21 and 22 to 42 days of life.

| Ingredients (%) | 1 – 21 days | 22 – 42 days |
| --- | --- | --- |
| Corn | 56,75 | 63,09 |
| Soybean meal | 35,17 | 28,09 |
| Soybean oil | 3,64 | 4,81 |
| Calcareous 34 Ca | 1,35 | 1,28 |
| NaCl | 0,34 | 0,31 |
| Sodium bicarbonate | 0,15 | 0,15 |
| Monobicaucium phosphate | 1,74 | 1,44 |
| DL-metionine | 0,31 | 0,27 |
| L-lisine | 0,24 | 0,26 |
| L-treonine | 0,11 | 0,10 |
| Vitamin premix^1^ | 0,1 | 0,1 |
| Mineral premix^2^ | 0,1 | 0,1 |
| Total | 100,00 | 100,00 |
|  |  |  |
| **Nutritional levels** | **Pre-initial** | **Initial** |
| Energy Kcal/kg | 3100 | 3250 |
| Protein % | 21,21 | 18,45 |
| Lisine dig % | 1,218 | 1,064 |
| Metionine dig % | 0,597 | 0,524 |
| Metionine + Cistine dig % | 0,876 | 0,774 |
| Treonine dig % | 0,791 | 0,689 |
| Phosphorus Disp % | 0,450 | 0,380 |
| Calcium Disp.% | 0,950 | 0,850 |
| Sodium % | 0,21 | 0,195 |

^1^Vitamin premix: Vitamin A 10.500.000 UI/kg; Vitamin D3 2.600.000 UI/kg; Vitamin E 20.000 UI/kg; Vitamin K3 2.500 mg/kg; Vitamin B1 1.900 mg/kg; Vitamin B2 6.500 mg/kg; Vitamin B6 2.900 mg/kg; Vitamin B12 17.000 mcg/kg; Niacin 40 g/kg; Pantothenic acid 12 g/kg; Folic acid 1.000 mg/kg; Biotin 65 mg/kg; Selenium 300 mg/kg, Butyl hydroxy toluene (BHT) 1.000 mg/kg.

^2^Mineral premix: Iron 55 g/kg; Cupper 9.000 mg/kg; Manganese 75 g/kg; Zinc 65 g/kg; Iodine 1.000 mg/kg.
