# Supplementary figures and images for: Different antibiotic growth promoters induce specific changes in the cecal microbiota membership of broiler chicken
Source: PLoS One. 2017 Feb 21;12(2):e0171642. doi: 10.1371/journal.pone.0171642 (PMC5319738; doi:10.1371/journal.pone.0171642)

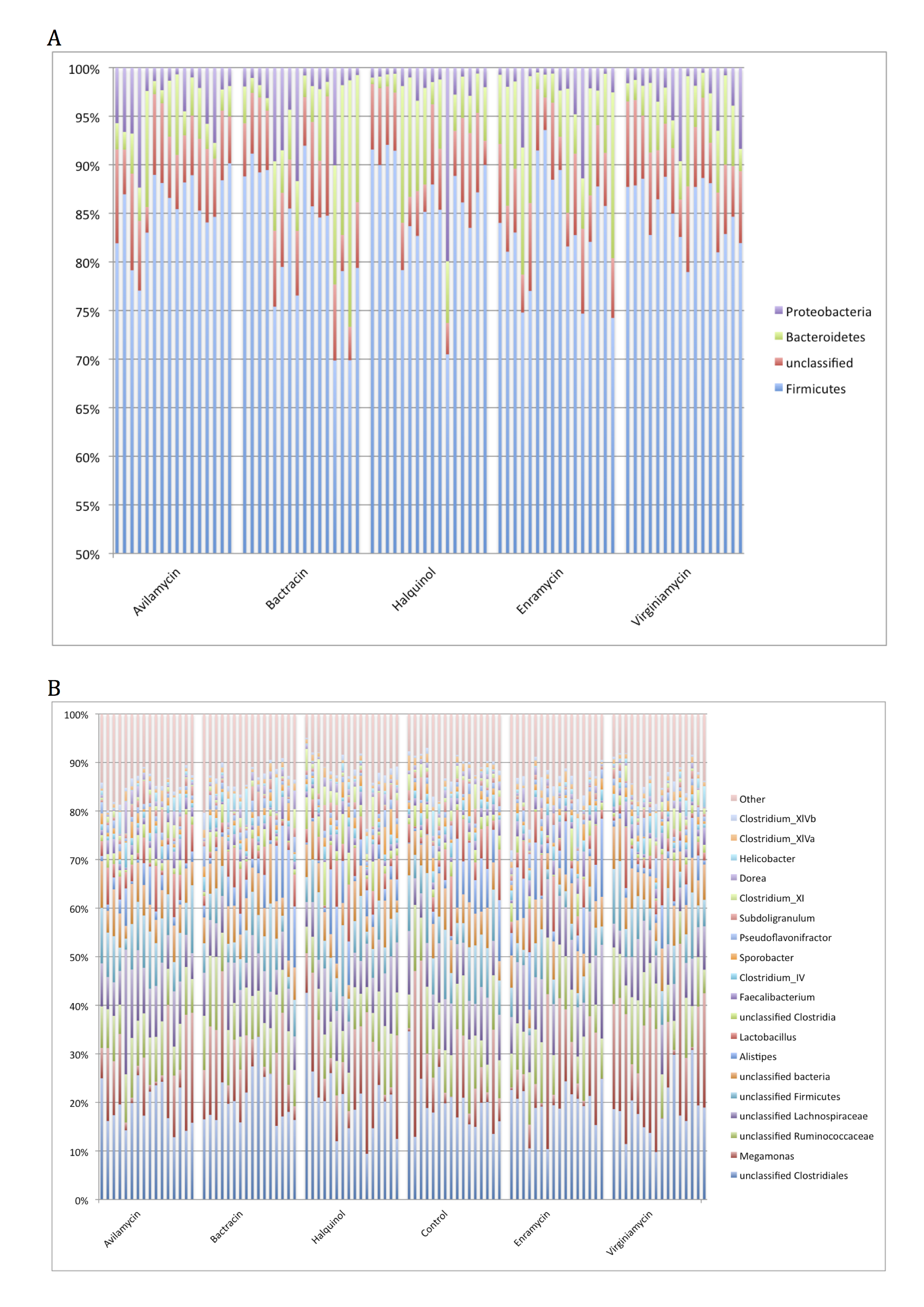

Supplement: S1 Fig — (TIFF) [file pone.0171642.s002.tiff]
